# Reintegrating Philosophy Into Postgraduate Psychiatry Training: Beyond Psychology Toward a More Humane Clinical Science

**DOI:** 10.7759/cureus.108826

**Published:** 2026-05-14

**Authors:** Dhiraj Raja

**Affiliations:** 1 Department of Psychiatry, Heritage Institute of Medical Sciences, Varanasi, IND

**Keywords:** clinical reasoning, medical education, patient-centered care, philosophy of psychiatry, psychiatry

## Abstract

In many settings, postgraduate psychiatry training now emphasizes neuroscience, evidence-based treatment, diagnostic criteria, and structured psychological models. These advances have improved psychiatric care in important ways. They have strengthened diagnosis, expanded treatment options, and improved scientific understanding of mental illness. At the same time, this increasing focus on structure and measurable symptoms can unintentionally narrow clinicians' understanding of patients. Psychiatry deals with more than just the brain. It also deals with meaning, identity, relationships, suffering, and lived experience.

Unlike many other medical specialties, psychiatry regularly requires clinicians to work with uncertainty, personal narratives, emotional complexity, and experiences that do not always fit neatly into diagnostic categories. In real clinical practice, patients rarely present as textbook cases. Clinicians must often decide whether a person’s suffering reflects a meaningful human response to life circumstances, a psychiatric disorder requiring intervention, or sometimes both.

In this editorial, I argue that postgraduate psychiatry training should integrate philosophy-informed and reflective approaches alongside scientific and evidence-based models. In this context, philosophy primarily encompasses phenomenology, ethics, philosophy of mind, reflective reasoning, and approaches that deepen clinicians' understanding of subjective experience. Earlier phenomenological perspectives emphasized the importance of understanding the patient’s inner world. At the same time, more recent pluralistic and neuroscience-informed models support a broader understanding of psychiatric illness that integrates biological, psychological, and social dimensions.

I also discuss how the rigid application of diagnostic frameworks can contribute to overmedicalization when clinicians fail to distinguish between distress and disorder carefully. Practical approaches such as reflective case discussions, formulation-focused supervision, phenomenological interviewing, ethics-based discussions, and narrative reflection can help integrate these perspectives into postgraduate training without requiring major curricular restructuring.

This approach does not reject scientific psychiatry. Instead, it complements scientific practice by strengthening reflective thinking, patient-centered understanding, ethical reasoning, and tolerance of clinical complexity. Psychiatry needs both scientific rigor and humane understanding if it hopes to remain fully connected to patients' lived experiences.

## Editorial

Psychiatry feels different from most other areas of medicine. In many fields, doctors identify a problem, measure it, and treat it directly. In psychiatry, clinicians work with something far less defined. Patients do not come with symptoms alone. They bring thoughts, emotions, beliefs, relationships, fears, memories, conflicts, and life experiences that often overlap and resist clear boundaries. At times, even patients struggle to explain what they feel or why they suffer.

In many settings, postgraduate psychiatry training now emphasizes neuroscience, evidence-based treatment, diagnostic criteria, and structured psychological models. These advances have strengthened psychiatric care in important ways, improving diagnosis, expanding treatment options, and deepening scientific understanding of mental illness. At the same time, the growing emphasis on structure and measurable symptoms can unintentionally narrow clinicians' understanding of patients. Psychiatry addresses more than biology alone. It also addresses meaning, identity, values, relationships, suffering, and lived experience.

In real-world clinical practice, patients rarely resemble textbook cases. They present with stories that are incomplete, layered, emotionally complex, and sometimes contradictory. Many young psychiatrists recognize this gap early in training. They understand the theoretical framework, but the reality of patient care often proves more complicated than expected. Clinical work regularly involves uncertainty, ambiguity, and difficult questions about distress, disorder, meaning, and responsibility.

We do not always teach how to manage this part of psychiatry.

This is where philosophy can help psychiatry.

In psychiatry, philosophy is not merely abstract theory. It encompasses phenomenology, ethics, philosophy of mind, the epistemology of diagnosis, reflective reasoning, and approaches that help clinicians think more carefully about subjective experience and human suffering. These perspectives help psychiatrists ask not only "What disorder does this patient have?" but also "What is this person experiencing?" and "What does this suffering mean in the context of this patient's life?"

Earlier work, such as the work by Laing, highlighted how deeply personal and subjective mental suffering can be [1]. Laing emphasized that people experience distress in ways that cannot always be reduced to simple diagnostic labels. His work focused attention on the patient's inner world and challenged purely external descriptions of illness. Modern psychiatry has moved beyond many of his conclusions, and clinicians should not interpret his work as a rejection of scientific psychiatry. However, the central concern he raised remains relevant: clinicians must recognize that similar symptoms can arise from very different lived experiences.

Jaspers further developed the distinction between explanation and understanding [2]. He argued that psychiatry should not limit itself to explaining symptoms through biological or psychological mechanisms alone but should also seek to understand the patient's subjective experience. This distinction remains relevant in modern psychiatric practice. Scientific explanation helps clinicians classify illness and guide treatment, while understanding helps interpret suffering within its personal, social, and emotional context. Patients do not always fit neatly into diagnostic categories, and clinicians often rely on reflective judgment when rigid frameworks alone are insufficient.

Scientific advances have clearly strengthened psychiatry, particularly through developments in neuroscience, psychopharmacology, genetics, and evidence-based care. However, clinical reality continues to show that no single model fully explains psychiatric illness.

A common example is the distinction between grief and depression. Both may involve sadness, crying, sleep disturbances, reduced appetite, emotional withdrawal, and difficulty concentrating. However, grief often arises in response to a meaningful loss and may gradually improve with time, support, and social connection. Depression, in contrast, may involve persistent hopelessness, loss of pleasure, marked functional impairment, suicidality, and broader neurovegetative symptoms that significantly interfere with daily functioning. In practice, the distinction is not always straightforward. Clinicians must consider severity, duration, context, risk, and functional impact before deciding whether intervention is necessary. Without careful judgment, there is a risk of overmedicalizing normal human distress by applying diagnostic frameworks too rigidly.

Szasz strongly challenged the medical model of psychiatry, arguing that mental illness is a social construct rather than a true disease [3]. Modern psychiatry does not accept this position in full, and substantial evidence supports the biological and neurobiological foundations of psychiatric disorders. However, Szasz's critique still serves an important reflective role. It reminds clinicians to think carefully about how psychiatric labels are applied and where the boundary lies between illness, distress, and socially unacceptable behavior. These historical critiques do not reject scientific psychiatry or endorse antipsychiatry positions. Instead, they encourage clinicians to use thoughtful diagnostic reasoning and pay closer attention to human experience.

More recent work supports a broader, more integrated understanding of psychiatric illness [4]. Kendler argues against rigid "biological vs. psychological" dichotomies and instead advocates a pluralistic understanding of causation. His model recognizes that psychiatric disorders emerge from continuous interaction among genetic vulnerability, neurobiology, personality, trauma, developmental experiences, relationships, and social context. Importantly, this pluralistic framework strengthens psychiatry without compromising scientific rigor. Instead, it strengthens psychiatry by aligning theoretical models more closely with what clinicians observe in real-world practice, where biological, psychological, and social influences constantly interact.

At the same time, neuroscience continues to strengthen the biological foundations of psychiatry [5]. Advances in genetics, neuroimaging, and psychopharmacology have identified consistent biological patterns linked to many psychiatric disorders. Insel's work highlights how neuroscience continues to refine understanding of conditions such as schizophrenia while also revealing the complexity of brain-behavior relationships. These advances reinforce psychiatry's place within medicine. However, neuroscience alone cannot fully explain meaning, suffering, identity, or subjective experience. Psychiatry, therefore, requires both scientific understanding and reflective interpretation.

These perspectives do not contradict one another. Instead, they point toward a more comprehensive model of psychiatric practice. Psychiatry does not need to choose between biology and human understanding. It requires both.

In daily clinical practice, this balanced approach manifests in simple yet important ways. Clinicians pause before assigning a diagnosis. They distinguish distress from disorder more carefully. They consider not only symptoms but also meaning, relationships, values, trauma, culture, and the patient's broader life context. When faced with uncertainty, they rely not only on diagnostic criteria but also on reflective judgment and ethical reasoning.

This approach also shapes clinicians' experience of their work. In some settings, when psychiatry is reduced to checklists, protocols, and symptom counting, clinical work can feel mechanical. In contrast, when clinicians engage more deeply with patients' experiences, their work often feels more meaningful and connected. This does not replace scientific psychiatry. It complements it by restoring the human dimension of care.

Importantly, achieving this balance does not require major restructuring of postgraduate training. Small, practical changes can help. Training programs can incorporate reflective case discussions, formulation-focused supervision, phenomenological interviewing exercises, ethics seminars, Balint-style discussion groups, and narrative reflection sessions. These approaches can help trainees tolerate uncertainty, strengthen reflective thinking, and more effectively integrate biological understanding with subjective patient experience. In many cases, educators can integrate these methods into existing academic discussions and supervision structures without imposing a major curricular burden.

Future educational research may help clarify whether reflective and philosophy-informed approaches improve communication skills, empathy, therapeutic alliance, diagnostic reasoning, and patient-centered care outcomes among psychiatry trainees. Further discussion is also needed on how to integrate these approaches into competency-based psychiatric education.

At its core, psychiatry involves sitting with another person to understand their experience. Scientific knowledge provides essential tools for diagnosis and treatment. But understanding suffering requires more than diagnostic criteria alone.

Psychiatry needs both scientific rigor and humane understanding to remain fully connected to patients' lived experiences.

A psychiatrist not only identifies symptoms but also seeks to understand the person behind those symptoms.

Conclusions

Psychiatry does not need to choose between science and philosophy. Both play important roles in understanding mental illness and in patient care. Modern training rightly emphasizes neuroscience, evidence-based treatment, and structured clinical models, but scientific knowledge alone cannot fully explain human suffering, meaning, or lived experience.

Even small efforts to include reflective and philosophy-informed thinking can strengthen clinical judgment, improve patient-centered understanding, and help clinicians engage more thoughtfully with complexity and uncertainty. Without this balance, psychiatry risks becoming efficient but emotionally limited. By integrating both scientific rigor and humane understanding, psychiatry can remain both clinically strong and deeply human.
